# Optimization of Citrus Pulp Waste-Based Medium for Improved Bacterial Nanocellulose Production

**DOI:** 10.3390/microorganisms12102095

**Published:** 2024-10-20

**Authors:** Carlotta Minardi, Davide Bersanetti, Essi Sarlin, Ville Santala, Rahul Mangayil

**Affiliations:** 1 Faculty of Engineering and Natural Sciences, Tampere University, 33100 Tampere, Finland; carlotta.minardi@kuleuven.be (C.M.); davide.bersanetti@aalto.fi (D.B.); essi.sarlin@tuni.fi (E.S.); ville.santala@tuni.fi (V.S.); 2 Department of Chemical Engineering, Chemical and Biochemical Reactor Engineering and Safety (CREaS @ De Nayer), KU Leuven, J. De Nayerlaan 5, 2560 Sint-Katelijne-Waver, Belgium; 3 Department of Bioproducts and Biosystems, Aalto University, 02150 Espoo, Finland

**Keywords:** *Komagataeibacter sucrofermentans*, citrus pulp waste, bacterial nanocellulose, Plackett–Burman, response surface methodology, baker yeast hydrolysate

## Abstract

Bacterial nanocellulose (BC) has attracted significant attention across a wide array of applications due to its distinctive characteristics. Recently, there has been increasing interest in leveraging waste biomass to improve sustainability in BC biogenesis processes. This study focuses on optimizing the citrus pulp waste (CPW) medium to enhance BC production using *Komagataeibacter sucrofermentans*. The screening of initial medium pH, yeast extract, CPW sugar and inoculum concentrations was conducted using the Plackett–Burman design, with BC yield (mgDW/gCPW) as the model response. The significant parameters, i.e., CPW sugars and yeast extract concentrations, were optimized using response surface methodology, employing a five-level, two-factor central composite design. The optimized CPW-based growth medium resulted in a final yield of 66.7 ± 5.1 mgDW/gCPW, representing a 14-fold increase compared to non-optimized conditions (4.3 ± 0.4 mgBC/gCPW). Material characterization analysis indicated that the produced BC showed high thermal stability (30% mass retained at 600 °C) and a crystallinity index value of 71%. Additionally, to enhance process sustainability, spent baker’s yeast hydrolysate (BYH) was assessed as a substitute for yeast extract, leading to a final BC titer of 9.3 ± 0.6 g/L.

## 1. Introduction

Extracellular production of cellulose has been reported in several bacterial species, among which the bacteria in the genus *Komagataeibacter* (formerly known as *Acetobacter* and *Gluconoacetobacter*) have been identified as the most efficient nanocellulose producers [1,2,3]. Unlike plant-derived cellulose, bacterial nanocellulose (BC) possesses exceptional attributes, such as high purity (absence of hemicellulose, lignin, and pectin), superior mechanical and physical properties, and benefits in biodegradability and biocompatibility [4]. Given these exceptional traits, BC has undergone comprehensive investigation for its multifaceted application [5,6]. While BC boasts versatility, its potential is curtailed by suboptimal production metrics. The literature highlights various bioprocesses and strain engineering endeavors aimed at enhancing BC production. However, BC production is conventionally conducted in rich Hestrin–Schramm (HS) medium, containing other carbon-containing components such as peptone and yeast extract (YE) that can contribute towards increased bioprocess costs [7]. In response to this challenge, there has been a growing interest in utilizing waste materials as carbon and nitrogen sources for the growth and BC synthesis by *Komagataeibacter* spp. [8,9,10] For instance, the citrus pulp waste (CPW), a by-product of industrial processing of *Citrus* spp., contains soluble sugars, cellulose, hemicellulose, and pectin, making it an excellent substrate for various biotechnological applications [11,12]. Furthermore, apart from its cost-effectiveness, the utilization of CPW avoids competition with food or feed production. Considering its nutrient composition, CPW represents a sustainable and promising biomass for BC production [13]. The potential and feasibility of valorizing citrus-based resources for BC production have already been explored in previous studies [14,15,16]. However, the emphasis of these investigations has primarily centered on biomass treatment and characterization of the synthesized biomaterial.

This study focuses on identifying the critical factors in a CPW-based medium to design an optimal growth medium for improved BC production. The medium components that imparted significant effects on BC biogenesis were selected through Plackett–Burman design. The Plackett–Burman approach is a useful technique for systematically exploring the effects of multiple parameters, allowing for the efficient screening of effective variables while eliminating non-effective ones [17,18]. The effective variables were further optimized using response surface methodology (RSM) based on central composite design (CCD). CCD allows the optimization of significant culture parameters and the analysis of their interactions by building a second-order quadratic model for the response variable (BC yield) without using a complete factorial experiment [19]. This approach allows us to minimize the number of trials and experiments while reliably identifying and optimizing the significant parameters. Contemplating the crucial significance of nanocellulose applications with respect to its physical properties, the biopolymer obtained from the optimized CPW-based medium was thoroughly examined using X-ray diffractometry (XRD), scanning electron microscopy (SEM), and thermogravimetric analysis (TGA). Additionally, spent backer yeast hydrolysate was tested as potential low-value nitrogen and nutrient sources in the optimized medium as a replacement for the commercially available YE.

## 2. Materials and Methods

### 2.1. Materials

*Komagataeibacter sucrofermentans* (DSM 15973) was purchased from DSMZ (Leibniz Institute DSMZ, Braunschweig, Germany). Peptone, YE, glucose, citric acid, nitrogen chloride and potassium chloride Cellulase from *Trichoderma reesei* and protease from *Bacillus* sp. were purchased from Merck (Espoo, Finland). Disodium hydrogen phosphate, sodium chloride, trehalose and agar were purchased from Fisher Scientific (Vantaa, Finland). Sodium hydroxide was purchased from VWR International Oy (Helsinki, FInland). The CPW was kindly provided by CNR-SCITEC (The Istituto di Scienze e Tecnologie Chimiche “Giulio Natta” (SCITEC) at the National Research Council, Milan, Italy).

The data analysis was conducted with Origin 2019b (OriginLab Corporation, Northampton, MA, USA). The statistical models were designed and analyzed using analysis of variance (ANOVA) in Minitab 20.0 (Statease Inc., Minneapolis, MN, USA).

### 2.2. Precultivation Conditions

To obtain the precultures, *K. sucrofermentans* was streaked on HS agar plates (g/L; 5 peptone, 5 YE, 2.7 Na_2_HPO_4_, 1.15 citric acid and 15 agar), containing 2% glucose and 1% cellulase, and incubated at 30 °C for 4–5 days. Single colonies were then inoculated in 50 mL flasks containing 20 mL of HS medium supplemented with 2% glucose (HS-Glucose) and statically incubated at 30 °C for 7 days. To free the bacterial cells entrapped within the BC, the produced pellicles were lysed overnight (O/N) with 1% cellulase. Subsequently, the cells were washed thrice with sterile PBS buffer (g/L; 8 NaCl, 0.2 KCl, 1.44 Na_2_HPO_4_, and 0.24 KH_2_PO_4_; pH 7.4) and resuspended in 5 mL of the buffer. This preculture suspension was used in subsequent experiments.

### 2.3. CPW Processing and Characterization

The dehydrated liquid fraction of the CPW was used as the substrate in this study. The fraction contained a total sugar concentration of 36% (55% purity) with a weight ratio of 1:3.27:5.78 between sucrose, glucose, and fructose, respectively, as analyzed at CNR-SCITEC using a gas chromatograph. Thus, to obtain the desired sugar concentration, dilutions with autoclaved Milli-Q (MQ) water were conducted. For instance, by resuspending 100 g of the biomass in 1 L of sterile MQ, a liquid fraction containing a total sugar concentration of ~20 g/L (2 g/L sucrose + 6.5 g/L glucose + 11.5 g/L fructose) was obtained. Depending on the biomass concentration, the pH range of the diluted CPW was between 3.5 and 4. The pH was adjusted using 5 M NaOH to the studied conditions (see 2.2 Plackett–Burman design). Following pH adjustments, the diluted CPW was centrifuged at 22,000× *g* for 15 min to remove the insoluble fraction. The supernatant, sterilized by autoclaving, was used as the substrate in subsequent experiments.

### 2.4. BC Processing and Material Characterization

BC pellicles were processed as mentioned in Cannazza et al. [6]. Briefly, after the incubation period, the BC pellicles were harvested and repeatedly washed in MQ water. The bacteria entrapped within the BC pellicles were inactivated by overnight (O/N) incubation in 0.5 M sodium hydroxide solution 60 °C. Following the incubation, the BC sheets were washed thoroughly with ultrapure MQ until neutral pH was attained and oven dried O/N on pre-weighed weighing boats at 60 °C. The dry weight (DW) was measured on an analytical balance (ES 220A, Precisa, Dietikon, Switzerland) and the yield was calculated using Equation (1). The oven-dried BC films were analyzed using SEM (Zeiss ULTRAPlus, Jena, Germany), XRD (Empyrean multipurpose diffractometer, Malvern Panalytical Ltd., Malvern, UK), and TGA (TG 209 F3 Tarsus, Netzsch-Gerätebau GmbH, Selb, Germany) as described in [1]. The crystallinity indices (CIs) were calculated from the peak area of the XRD data using the Segal method in Origin 2019b [2,3,4]. For cross-sectional SEM images, the BC films were cryofractured under liquid nitrogen and attached to holders with conductive carbon cement. The samples were coated with a thin carbon layer for electrical conductivity before imaging.

### 2.5. Preliminary Assessment of BC Production from CPW

To assess BC production from CPW, *K. sucrofermentans* was inoculated in triplicates, using the preculture suspension (detailed in Supplementary Methods), into 10 mL of sterile CPW solution containing a sugar concentration of 20 g/L. The initial test was conducted through static cultivation at 30 °C, devoid of any growth supplements. Both BC and high-performance liquid chromatography (HPLC) samples were collected after 7 and 14 days of cultivation. The BC sheets were purified as explained in the Supplementary Methods and the HPLC samples were analyzed as explained in Section 2.6. The yield was calculated using the following equation:

(1) $$Yield\;(mg/g)=\frac{mgDW\left(BC\right)}{gSubstrate\;(CPW)}\;$$

where mgDW (BC) is the dry weight (g) of the BC sheet and the gSubstrate (CPW) is the mass of CPW.

### 2.6. Plackett–Burman Design

The design model was employed to identify the factors in CPW-based medium that imparted significant influence on *K. sucrofermentans* growth and BC biogenesis capacity. In the design, BC yield (Equation (1)) was chosen as the model response and the sugar concentration (CPW sugars), YE (YE, nitrogen source), initial pH (ipH) and inoculation concentration (OD_600nm_) were chosen as variables. The factors were selected based on related literature and the preliminary results from this study [19,20,21]. Each experimental run was conducted in triplicates at 30 °C in static conditions for 14 days. At the end of the cultivation, both BC and HPLC samples were collected and analyzed as mentioned in Section 2.10. The full experimental design can be found in Table 1. Control runs were also performed in triplicates on MQ containing respective YE concentrations to assess the contribution of the carbon in the YE on the BC yield.

### 2.7. Central Composite Design (CCD)

To optimize BC production from CPW-based medium, the statistically significant factors identified from Plackett–Burman design (YE and CPW sugars) were further investigated using central composite design (CCD) [17,22]. The factors that did not demonstrate statistical significance were held at predetermined levels based on their impact on BC yield. Hence, the initial pH and inoculum concentration was kept at 5 and 0.2 OD_600nm_, respectively. A 13-run CCD including 5 centre points, 4 axial points (±α=2K) and 4 factorial points ($\pm 1=2^{K}$) (K=number of factors) was performed [23]. To investigate the effect of the analyzed values and estimate the quadratic effect of the model, the factors selected were tested at five levels (−α, −1, 0, +1, +α; with α = 1.41). Based on the Plackett–Burman design results, the central values of YE and CPW sugar concentrations were kept at 5 g/L and 20 g/L, respectively. The full experimental design is presented in Table 2. The cultivations were carried out in triplicates for 14 days in static conditions at 30 °C. The obtained BC pellicles were processed as explained in Section 2.4. BC yield (Equation (1)) was used as the model response. The experimental results were then fitted to a second-order polynomial equation by multiple regression analysis. For a two-factor CCD, the fitted response surface model (RSM) is represented by the following equation:

(2) Y=−28.70+6.02A+5.717B−0.28AA−0.1151BB−0.0539AB

where the model variables A and B are YE and CPW sugar concentrations, respectively, and the response Y represents the BC yield. Additionally, to analyze the sugar consumption profile, HPLC samples were taken at the beginning and at the end of the 14 days of cultivation, as explained in Section 2.10.

### 2.8. Validation Experiment

The validation experiments were carried out using the CPW medium that had been optimized as per the predictions made using Equation (2). The composition of the optimal CPW medium includes a CPW sugar concentration of 22.7 g/L, 8.6 g/L of YE and an initial pH of 5. The static cultivations were conducted in triplicates for 14 days at 30 °C using an inoculum concentration of 0.2 OD_600nm_.

### 2.9. Baker’s Yeast Hydrolysate Preparation and Characterization

The feasibility of spent yeast hydrolysate as an alternative nitrogen source to the YE in optimized CPW medium was tested, using baker’s yeast procured from a local store in Tampere, Finland. The BYH was prepared using enzymatic hydrolysis and thermal pretreatment methods as described in Luo et al. (2020) [24]. To identify the appropriate baker’s yeast hydrolysate (BYH) concentration to include in the optimized medium, varying concentrations of BYH were tested using the Bradford assay kit (BioRad, Hercules, CA, USA) to reach the protein content corresponding to that contributed by the YE [25]. To detect the presence of sugar components, BYH was analyzed using HPLC (Section 2.10).

### 2.10. HPLC Analysis

To analyze the substrate consumption profile, HPLC samples were centrifuged at 22,000× *g* for 5 min and filtered with a 0.2 µm filter into a standard HPLC sample vial. If not immediately analyzed, the samples were stored at −20 °C. The samples were analyzed using a Shimadzu HPLC equipped with Rezex RHM-Monosacharide H+ column (300 × 7.8 mm; Phenomenex, Torrance, CA, USA, SIL-20AC HT autosampler (Shimadzu, Kyoto, Japan), RID-10A refractive index detector (Shimadzu, Japan), and 5 mM H_2_SO_4_ as the mobile phase. Using the HPLC data, the substrate utilization was calculated using the following equation:

(3) $$Substrate\;Utilisation\;\left(\%\right)=100-\left(\frac{Ci}{Cf}\times 100\right)$$

where Ci refers to the initial total sugar concentration in the culture medium, and Cf to the total sugar concentration at the end of the cultivation.

## 3. Results and Discussion

### 3.1. Preliminary Assessment of BC Production on CPW

The preliminary BC production tests were conducted in a CPW medium (containing a sugar concentration of 20 g/L) devoid of YE. After 7 days of static incubation at 30 °C, *K. sucrofermentans* yielded 4.3 ± 0.4 mgBC/gCPW (BC titer, 0.43 ± 0.04 g/L), which slightly increased to 4.7 ± 0.5 mgBC/gCPW (BC titer, 0.5 ± 0.1 g/L) after 1 week of prolonged incubation. The yield observed in this study was relatively low when compared to values reported in the literature [16,26]. This could be attributed towards the negative impact of the acidic pH of the CPW-based medium on BC production [16]. Furthermore, the limited availability of a nitrogen source in CPW medium (devoid of YE) may be the reason for the low BC production. HPLC analysis demonstrated distinct levels of sugar consumption in CPW medium (Figure S1). After 14 days of cultivation in CPW medium, *K. sucrofermentans* had an overall CPW utilization of 29%. As anticipated with *Komagataeibacter* spp. cultivated in a glucose-containing medium, the conversion of glucose to gluconate, facilitated by glucose dehydrogenase, led to a gradual decrease in pH, reaching a value of 2.5 after 14 days. Given the BC yield obtained, a cultivation period of 14 days was selected for subsequent tests. To mitigate the adverse impacts of the low initial pH, pH adjustment of CPW-based medium was performed using 5 M NaOH. Waste biomasses, notably CPW, have been reported to harbour bioactive compounds like flavonoids, which have antimicrobial activity [13,27,28]. The potential presence of flavonoids in the CPW used in this study, identified during pH adjustments (Figure S2), could also serve as a plausible explanation for the observed low BC yield in this initial test.

### 3.2. Plackett-Burman Screening

Hypothesizing that the low yield could be attributed to the low nitrogen content in the medium, a concentration of 5 g/L YE (used in conventional HS medium) was selected. The impacts of the CPW sugar and YE concentrations, initial pH of the media, and initial inoculum concentration on bacterial cellulose production by *K. sucrofermentans* were investigated using Plackett–Burman design. Table 1 presents the Plackett–Burman experimental design and results. BC production was observed in all 12 experimental runs, with the highest yield (34.0 ± 4.6 mgBC/gCPW) in Run 2 (BC titer, 3.4 ± 0.6 g/L) and the lowest (7.2 ± 1.1 mg/g) in Run 12 (BC titer, 1.4 ± 0.2 g/L). The final medium pH ranged from 2.5 to 3, indicating the expected conversion of glucose into gluconate during the 14-day incubation period. The ANOVA analysis of the Plackett–Burman design is presented in Table 3. Both CPW sugars and YE were identified as statistically significant variables (*p*-value < 0.05) with a *p* value of 0.03 and 0.0, respectively. The regression equation constructed for this model with BC yield was as follows:

(4) BC yield=28.8−0.347A−1.81B+31.4C+2.957D 

where A is the CPW sugar concentration; B is the initial medium pH; C is the inoculum concentration and D, is the YE concentration.

**Figure 1:**
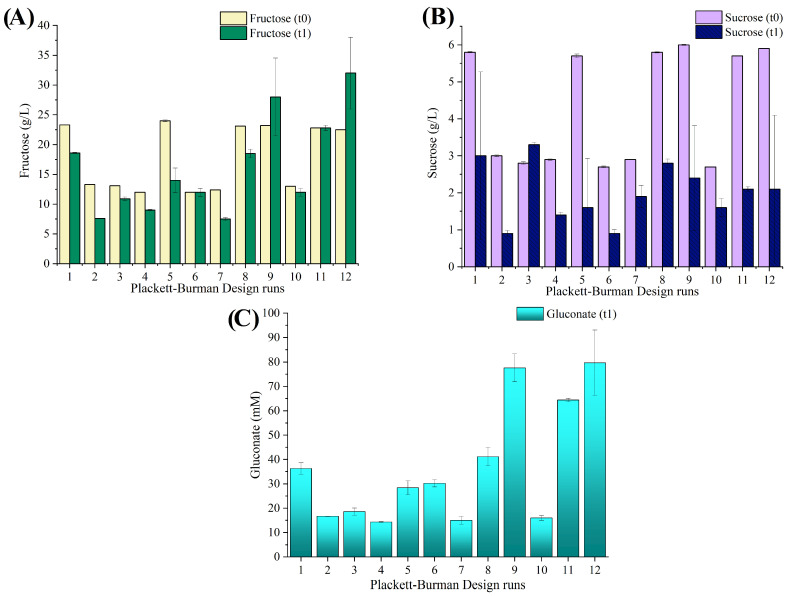
Fructose (**A**) and sucrose (**B**) utilization, and gluconic acid (**C**) production profiles of *K. sucrofermentans* grown from Plackett–Burman design runs. The initial and final concentrations are presented in the graph as t0 and t1, respectively. The averaged values and standard deviations (indicated as error bars) from triplicate cultivations are presented. In some cases, the error bars are smaller than the edge of the plot bar.

The pareto charts of the tested factors on BC yield are presented in the Supplementary Materials (Figure S3). Considering that proteins make up 8–14% of the dry weight of bacteria and the crucial role nitrogen sources play in bacterial metabolism, it was advisable to maintain higher levels of YE in subsequent optimization tests to achieve improved BC production, especially considering its significant positive effect observed in the Plackett–Burman runs. On the other hand, as CPW sugar concentration exerts a negative effect on BC yield, the model suggests keeping the variables at a lower level. The initial pH of the medium and the inoculum concentration did not exhibit a significant effect on BC yield. Therefore, according to the surface plots, an initial pH of 5 and inoculum concentration at 0.2 OD_600nm_ were maintained. HPLC analysis of the 12 runs (Figure 1) aligns with the findings observed during the preliminary test (Section 3.1) with some variations. Glucose underwent complete metabolization, while sucrose was partly consumed. Among the carbon sources present in the CPW, fructose exhibited the lowest consumption levels. The notable high yield in Run 2 was reinforced by the observed high total sugar utilization (68%), as well as the lowest recorded gluconate concentration (16.7 ± 0.1 mM) at the conclusion of the 14-day cultivation period.

### 3.3. Central Composite Design

CCD was employed to determine the optimized values of the factors that exhibited statistically significant positive effects on BC yield, as identified through the Plackett–Burman design. The CCD experimental design, parameters and results are presented in Table 2. Among the tested runs, the highest BC yield (61.0 ± 7.3 mgBC/gCPW) was observed in Run 13 (BC titer, 6.1 ± 0.7 g/L), and the lowest (25.0 ± 1.8 mgBC/gCPW) in Run 1 (BC titer, 1.0 ± 0.1 g/L). Multiple regression analysis was applied to the experimental data and a second-order polynomial model was obtained (Equation (2)). The statistical significance of the model and its parameters was assessed using ANOVA at a confidence level of 95% (Table 4). The calculated *p* value for the model was lower than 0.05, indicating its high statistical significance. Furthermore, the S-Value (3.4), R2 (95.18%), and adjusted R2 (91.74%) collectively indicate a strong alignment between the experimental data and the model, leaving only 4.8% of the sample variation unaccounted for.

**Figure 2:**
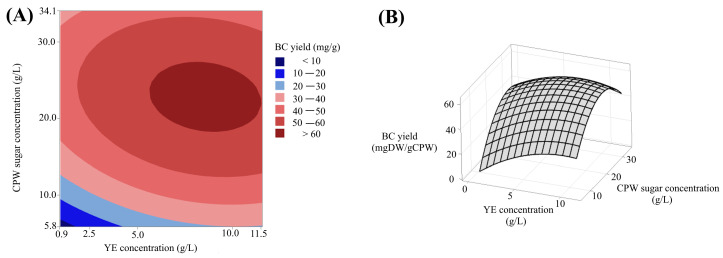
(**A**) Two-dimensional contour and (**B**) three-dimensional surface plots showing the effects of CPW sugar and YE concentrations on BC yield.

The normal probability plot of the standardized effects indicates statistically significant effects of YE and CPW sugars on BC yield (Figure S4A). Additionally, the internally studentized residuals (Figure S4B) suggest that the errors follow a normal distribution, indicating the errors in the experimental data are independent from one another. Hence, it is possible to refuse the hypothesis that the observed deviations are due to chance. We also observed that both factors (CPW sugars and YE) and their square of terms (AA and BB) have a statistically relevant impact on *K. sucrofermentans* BC production in the CPW-based medium. The statistical significance of these factors is validated by their respective P Values, all of which are lower than 0.05. Furthermore, the ANOVA analysis allows us to confidently dismiss the statistical significance of the interaction between YE and CPW sugar concentration (*p* value YE*CPW sugars = 0.3), confirming their independence. Figure 2A,B illustrate the two-dimensional contour curve and corresponding three-dimensional response surface plots on CPW sugars and YE concentration. Elliptical contours are obtained when a substantial interaction among the tested variables towards the targeted response is predicted [29]. These plots reveal that the region associated with the escalating concentrations of CPW yielded detrimental effects on BC production (with the highest predicted yield for concentrations ranging between 18.0 and 27.0 g/L), whereas YE imparted positively (with concentrations ranging between 6.0 and 11.0 g/L).

**Figure 3:**
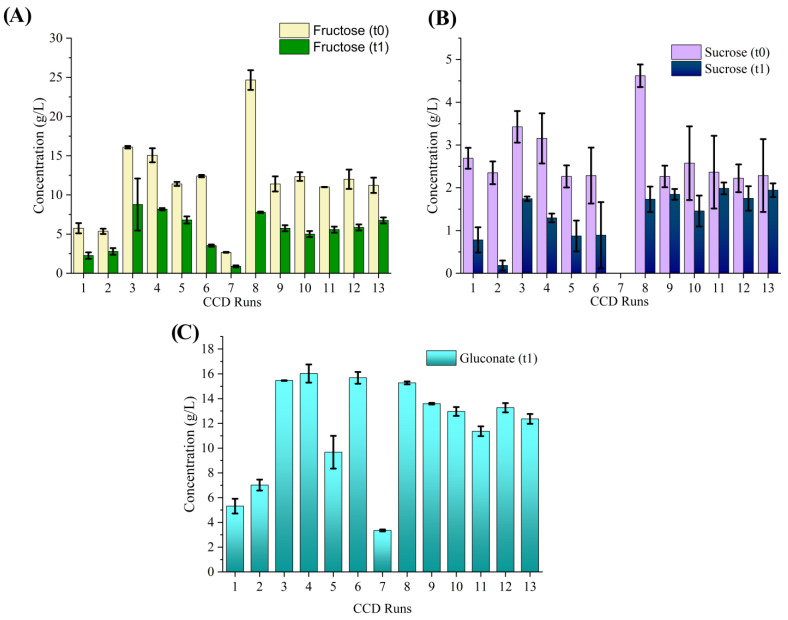
Fructose (**A**) and sucrose (**B**) utilization, and gluconic acid (**C**) production profiles of *K. sucrofermentans* grown from CCD runs. The initial and final concentrations are presented in the graph as t0 and t1, respectively. The averaged values and standard deviations (indicated as error bars) from triplicate cultivations are presented. In some cases, the error bars are smaller than the edge of the plot bar.

In comparison to the Plackett–Burman runs (Figure 1), CCD runs showed a lower final gluconate concentration (Figure 3), which may be a reason for the relatively higher final medium pH (Table 2) after 14 days of cultivation. Additionally, compared to the Plackett–Burman design runs, the CCD runs showed a higher fructose consumption. The CCD run that exhibited the highest yield (Run 13) displayed a sugar utilization of 62.3% and a gluconate concentration of 12.4 ± 0.4 mM.

### 3.4. Model Validation

**Figure 4:**
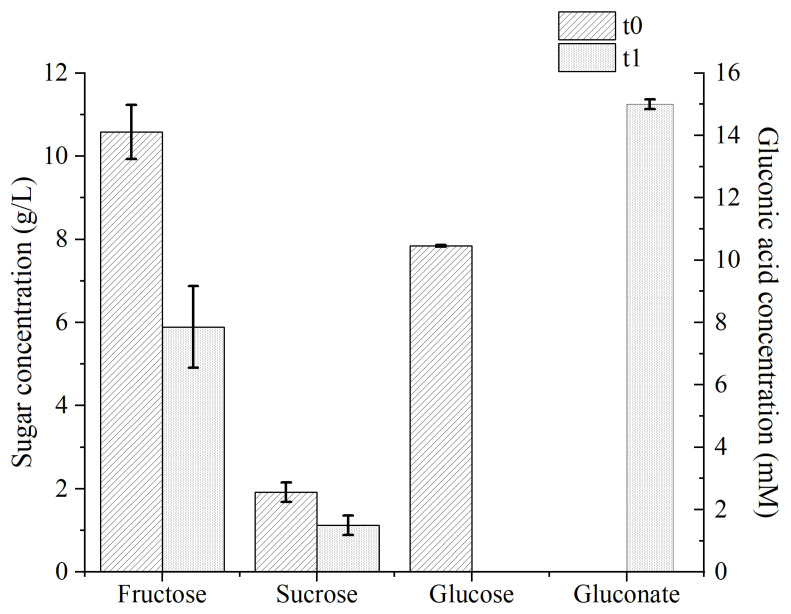
CPW sugar utilization and gluconic acid production profiles of *K. sucrofermentans* grown in the optimized CPW-based medium. The initial and final concentrations are presented in the graph as t0 and t1, respectively. The averaged values and standard deviations (indicated as error bars) from triplicate cultivations are presented. In some cases, the error bars are smaller than the edge of the plot bar.

Using Equation (2), it was possible to identify the optimal values of YE and CPW sugar concentrations predicted to maximize BC production. A maximum yield of 62.4 mgBC/gCPW (95% CI between 58.2 and 66.5 mgBC/gCPW; 95% PI between 53.1 and 71.6 mgBC/gCPW) was predicted, using 8.7 g/L and 22.8 g/L of YE and CPW sugar concentrations, respectively. To validate the model, the optimal values of both the factors were tested in triplicates. With the optimized CPW-based medium, a BC yield of 66.7 ± 5.1 mgBC/gCPW and a titer of 6.7 ± 0.5 g/L (corresponding to a 14-fold increase with respect to the preliminary tests) was obtained. Furthermore, the observed values aligned well within the model prediction interval (PI) and confidence interval (CI). Following the 14-day cultivation period, the final medium pH reached 4.0. The HPLC results (Figure 4) were consistent with the CCD run data. Glucose was entirely metabolized, whereas sucrose and fructose displayed partial metabolism, approximately reaching a half of their respective initial concentrations. The total sugar utilization was 65.5% and gluconate reached a final concentration of 15.0 ± 0.2 mM. The BC production achieved within the scope of this study outperforms the findings documented in the existing literature under comparable conditions. For instance, Kurosumi et al. (2009) have reported a BC titer of 5.9 g/L from *Acetobacter xylinum* NBRC 13693 following a 14-day cultivation period in orange juice-based growth medium containing 5 g/L YE and 20 g/L peptone as nitrogen sources [30]. Similarly, a BC titer of 5.7 ± 0.5 g/L was reported by Fan et al. (2016) from *K. xylinus* CICC No. 10529 grown in citrus peel and pomace enzymolysis medium containing 4.0 g/L YE, 4.0 g/L peptone and 0.8% ethanol [26]. In another study, a BC titer of 3.2 ± 0.70 g/L was obtained from *Gluconacetobacter xylinus* BCRC 12334 after an 8-day static cultivation in an acetate buffered orange peel hydrolysate that contained 5 g/L of YE and peptone as nitrogen sources [12].

### 3.5. Material Characteraization

**Figure 5:**
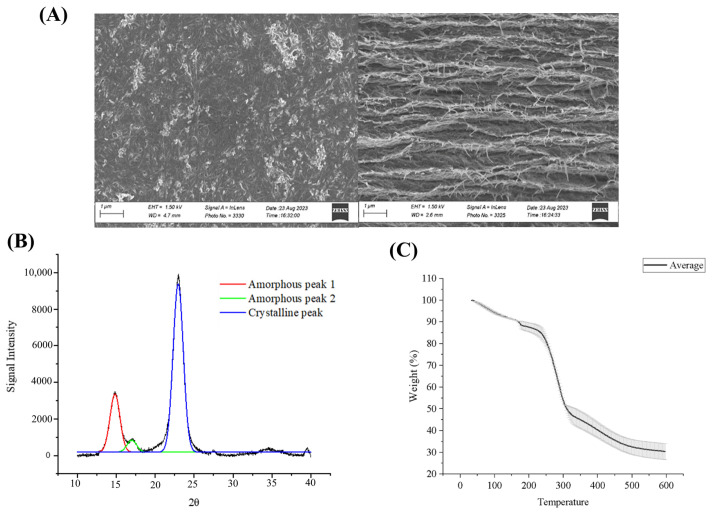
Characterization of bacterial nanocellulose produced from the validated CPW-based medium including a CPW sugar concentration of 22.7 g/L, 8.55 g/L of YE and an initial pH of 5. Static cultivations were conducted in triplicates for 14 days at 30 °C using an inoculum concentration of 0.2 OD_600nm_. (**A**) shows the surface (**left**) and cross-sectional (**right**) SEM images. (**B**) displays the XRD analysis deconvolution graph. The areas of the three peaks (amorphous and crystalline cellulose) were analyzed from the raw data (in black) and used to calculate the crystallinity index. (**C**) illustrates the thermogravimetric profile. The graph reports averaged values and standard deviations (indicated as error bars) from triplicate cultivations of the optimized medium (validation experiment).

The SEM image (Figure 5A) indicated that the BC produced from the optimized medium showed a similar crisscrossed nanofibrillar arrangement that is conventionally observed in BC films [2,26]. The XRD spectra (Figure 5B) revealed three dominant diffraction peaks at 14.8°, 16.9° and 22.9° that present the cellulose allomorphs. The crystallinity index value of 71.3% ± 0.3, calculated using the peak deconvolution method, was comparable to that reported in the literature for *K. sucrofermentans* [31,32]. The TGA curve (Figure 5C) presents the mass loss within the BC film while subjected to the studied temperature ranging between 30 and 600 °C. The observed gradual loss of mass (12%) at temperature ranges of 30–180 °C became drastic with an increase in temperature (43% at 330 °C) resulting in a final weight loss of 70%.

### 3.6. Replacing YE with Baker’s Yeast Hydrolysate

The Bradford assay identified that 70 g/L of BYH corresponded to the amino acid content of the YE in the optimized CPW medium. By replacing YE with BYH in the optimized CPW medium, a final BC titer of 9.3 ± 0.6 g/L was obtained, corresponding to a 1.5-fold increase compared to the result obtained from the validation experiment (6.7 ± 0.5 g/L). HPLC analysis of initial and end-point *K. sucrofermentans* samples cultivated in BYH indicated the presence of trehalose, a sugar component generally observed in yeast hydrolysate preparations (Figure S5) [33,34]. Although the trehalose utilization cannot be confirmed due to overlapping sucrose peaks (Figure S5), we hypothesize that the observed increase in BC titer could be attributed to the utilization of trehalose or other growth promoting compounds in the BYH.

## 4. Conclusions

The present study demonstrates the capability of *K. sucrofermentans* to produce BC from citrus processing waste. Utilizing the Plackett–Burman design and CCD, the culture conditions (inoculum concentration, 0.2 OD_600nm_; initial medium pH, 5) and the growth medium components (CPW sugars, 23.0 g/L; YE 8.7 g/L) were optimized to maximize the BC yield. YE and the CPW sugar concentration were found to have a significant impact on BC production and the current study was successful in improving the BC yield from 4.7 ± 0.5 mgBC/gCPW (BC titer, 0.5 ± 0.1 g/L) to 67.0 ± 5.1 mgBC/gCPW (BC titer, 6.7 ± 0.5 g/L, corresponding to a 14-fold increase), outperforming the previous reports documented in the literature obtained under comparable conditions. Although inclusion of BYH in the CPW-based medium improved BC production, reaching a BC titer of 9.3 ± 0.6 g/L, future studies are necessary to further investigate and confirm the feasibility of BYH as an alternative to the commercial YE for industrial BC production.

## Figures and Tables

**Table 1:** Plackett–Burman experimental design with four independent variables along with the model response BC yield (mgDW/gCPW). The averaged values ± standard deviations from triplicate cultivations after 14 days of static incubation at 30 °C are presented. Factors A, B, C and D correspond to CPW sugars (g/L), initial pH, inoculum concentration (OD_600nm_) and YE (g/L).

	CPW Sugar Concentration (A)		Initial pH (B)		Inoculum Concentration (C)		YE Concentration (D)		BC Yield (mgDW/gCPW) ^a^	
Runs	g/L	Level	pH	Level	OD_600nm_	Level	g/L	Level		Final pH
1	40	+1	7	+1	0.05	−1	5	+1	12.0 ± 4.5	2.8
2	20	−1	7	+1	0.2	+1	5	+1	34.0 ± 4.6	3
3	20	−1	7	+1	0.2	+1	0	−1	13.0 ± 0.9	2.8
4	20	−1	5	−1	0.05	−1	5	+1	26.5 ± 2.8	3
5	40	+1	5	−1	0.2	+1	5	+1	31.0 ± 10.9	2.5
6	20	−1	5	−1	0.05	−1	0	−1	18.0 ± 4.6	2.5
7	20	−1	5	−1	0.2	+1	5	+1	31.0 ± 1.5	3
8	40	+1	7	+1	0.05	−1	5	+1	23.5 ± 3.2	2.5
9	40	+1	7	+1	0.2	+1	0	−1	8.0 ± 2.2	3
10	20	−1	7	+1	0.05	−1	0	−1	12.5 ± 2.3	3
11	40	+1	5	−1	0.2	+1	0	−1	11.0 ± 0.6	2.5
12	40	+1	5	−1	0.05	−1	0	−1	7.0 ± 1.1	2.8

^a^ The BC yield and the standard deviation values were the means obtained from triplicate experiments and have been rounded to the nearest integer.

**Table 2:** Two-factor central composite experimental design table presenting the model response (BC yield) and the final medium pH after 14 days of static incubation at 30 °C. The averaged values ± standard deviations from triplicate cultivations are presented. Factors A and B correspond to YE (g/L) and CPW sugars (g/L).

	YE		CPW Sugars		BC Yield (mgDW/gCPW) ^a^	
Runs	A (g/L)	Code A	B (g/L)	Code B		Final pH
1	2.5	−1	10	−1	25.0 ± 1.8	5
2	10	+1	10	−1	43.0 ± 2.0	6.5
3	2.5	−α	30	+1	50.0 ± 2.1	3.5
4	10	+1	30	+1	58.0 ± 6.4	4
5	0.9	0.9	20	0	46.0 ± 4.4	3.5
6	11.6	+α	20	0	58.0 ± 1.5	6
7	5	0	5.9	−α	26.0 ± 2.0	5.5
8	5	0	34.1	+α	43.0 ± 2.8	4
9	5	0	20	0	54.0 ± 3.6	4
10	5	0	20	0	57.0 ± 6.1	4
11	5	0	20	0	59.0 ± 3.1	4.5
12	5	0	20	0	56.0 ± 6.4	4
13	5	0	20	0	61.0 ± 7.3	4

^a^ The mean BC yield values obtained from triplicate experiments have been rounded to the nearest integer.

**Table 3:** Results of ANOVA analysis for the Plackett–Burman design.

Source	df	Adj SS	Adj MS	*F* Value	*p* Value	Coefficient (Coded)	SE_coefficient_ (Coded)
Model	4	906.53	226.63	11.71	0.003		
Linear	4	906.53	226.63	11.71	0.003		
CPW sugars	1	144.91	144.91	7.48	0.029	−3.48	1.27
Initial medium pH	1	39.24	39.24	2.03	0.198	−1.81	1.27
Inoculum concentration	1	66.74	66.74	3.45	0.106	2.36	1.27
YE	1	655.64	655.64	33.86	0.001	7.39	1.27
Error	7	135.53	19.36				
Lack-of-fit	6	69.41	11.57	0.17	0.946		
Pure error	1	66.13	66.13				
Total	11	1042.06					

df, degree of freedom; Adj SS, adjusted sum of squares; Adj MS, adjusted mean square; SE_coefficient_, Standard error of coefficient S-value, 4.40; R^2^, 86.99%; adjusted R^2^, 79.56%.

**Table 4:** Details of the second-order polynomial model constructed using CCD.

Source	DF	Adj SS	Adj MS	*F* Value	*p* Value
Model	5	1666.43	333.287	27.67	0.000
Linear	2	696.88	348.442	28.93	0.000
YE	1	238.18	238.177	19.77	0.003
CPW (sugars)	1	458.71	458.708	38.08	0.000
Square	2	940.83	470.415	39.06	0.000
YE*YE	1	89.89	89.891	7.46	0.029
CPW sugars*CPW sugars	1	915.85	915.846	76.04	0.000
Two-way interaction	1	17.28	17.279	1.43	0.270
YE*CPW sugars	1	17.28	17.279	1.43	0.270
Error	7	84.31	12.044		
Lack-of-Fit	3	52.46	17.486	2.20	0.231
Pure error	4	31.85	7.963		
Total	12	1750.74			

df, degree of freedom; Adj SS, adjusted sum of squares; Adj MS, adjusted mean square. S-value, 3.47; R^2^, 95.18%; adjusted R^2^, 91.74%; predicted R^2^, 76.54%.

## Data Availability

All the data procured from this study can be found in the manuscript or in the accompanying Supplementary Materials. The raw data are available upon request.

## Supplementary Materials

The following supporting information can be downloaded at https://www.mdpi.com/article/10.3390/microorganisms12102095/s1, Figure S1: Preliminary BC production tests by K. sucrofermentans on CPW containing 20 g/L sugar total sugar content; Figure S2: pH dependent colour changes in CPW solution with a 20 g/L sugar concentration; Figure S3: Pareto chart of the standardised effect for the Plackett-Burman design factors (A, CPW sugars; B, ipH; C, inoculum concentration; D, YE) on BC yield, showing the statistical significance of CPW sugars and yeast extract; Figure S4: Fructose (A) and sucrose (B) utilisation, and gluconic acid (C) production profiles of K. sucrofermentans grown from Plackett-Burman design runs; Figure S5: Response surface regression analysis of central composite design (CCD).
