# Supplementary material for: Optimization of Citrus Pulp Waste-Based Medium for Improved Bacterial Nanocellulose Production
Source: Microorganisms. 2024 Oct 20;12(10):2095. doi: 10.3390/microorganisms12102095 (PMC11510694; doi:10.3390/microorganisms12102095)
Supplement: Supplementary file 1 [file microorganisms-12-02095-s001.zip › microorganisms-3240354-supplementary.pdf]

Supplementary figures

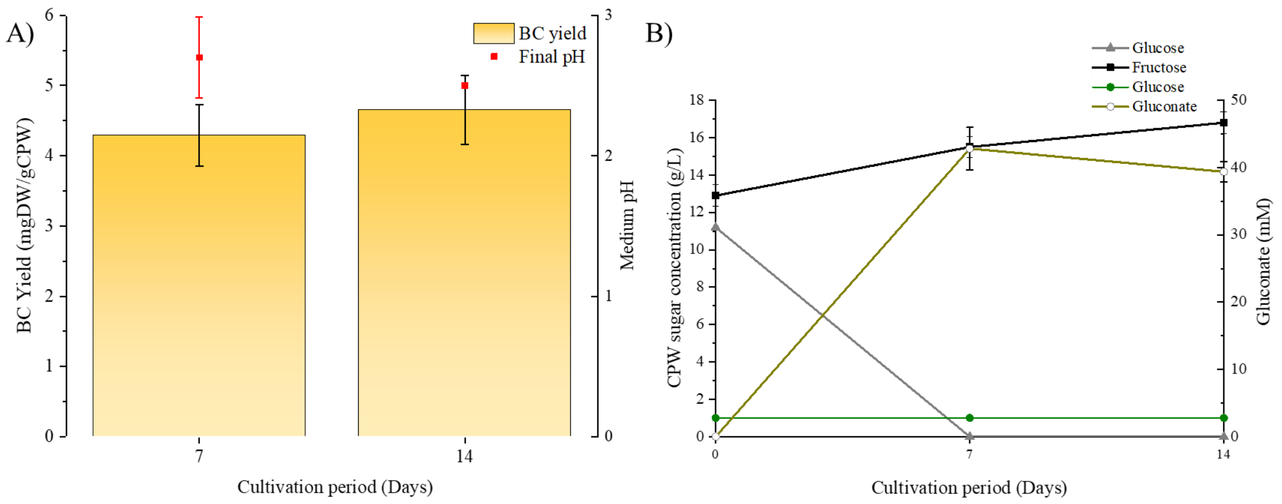

Figure S1. Preliminary BC production tests by *K. sucrofermentans* on CPW containing 20 g/L sugar total sugar content. A) BC yield (mgBC/gCPW) and final medium pH after 7 and 14 days of cultivation in CPW medium. B) Sugar consumption and gluconic acid production profile of *K. sucrofermentans* after 7 and 14 days of cultivation. The averaged values and standard deviations (error bars) from triplicate cultivations are presented. In some cases, the error bars are smaller than the symbols.

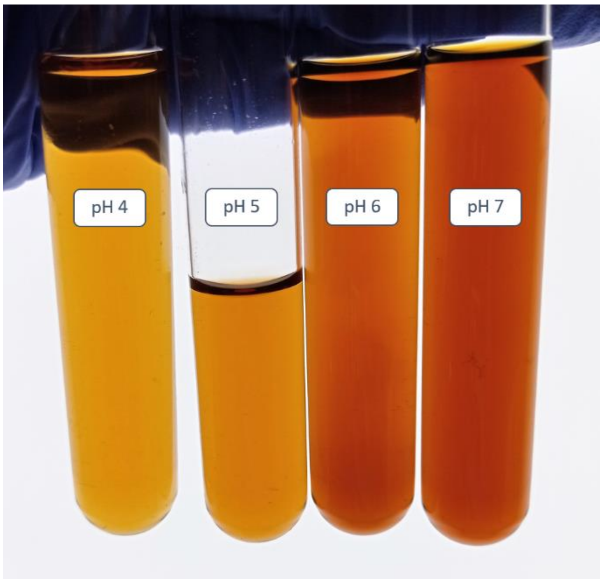

Figure S2. pH dependent colour changes in CPW solution with a 20 g/L sugar concentration. The pH was adjusted adding 5M NaOH and the colour change highlights the presence of flavonoids (anthocyanins) in the solution.

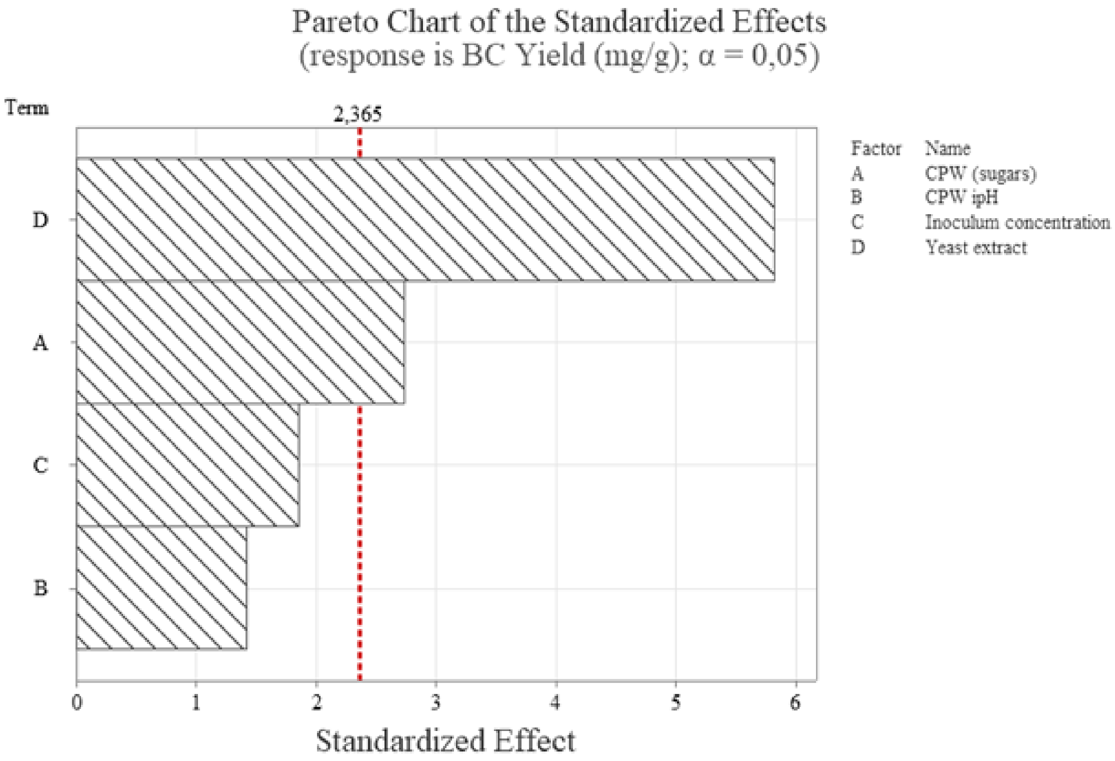

Figure S3. Pareto chart of the standardised effect for the Plackett-Burman design factors (A, CPW sugars; B, ipH; C, inoculum concentration; D, YE) on BC yield, showing the statistical significance of CPW sugars and yeast extract.

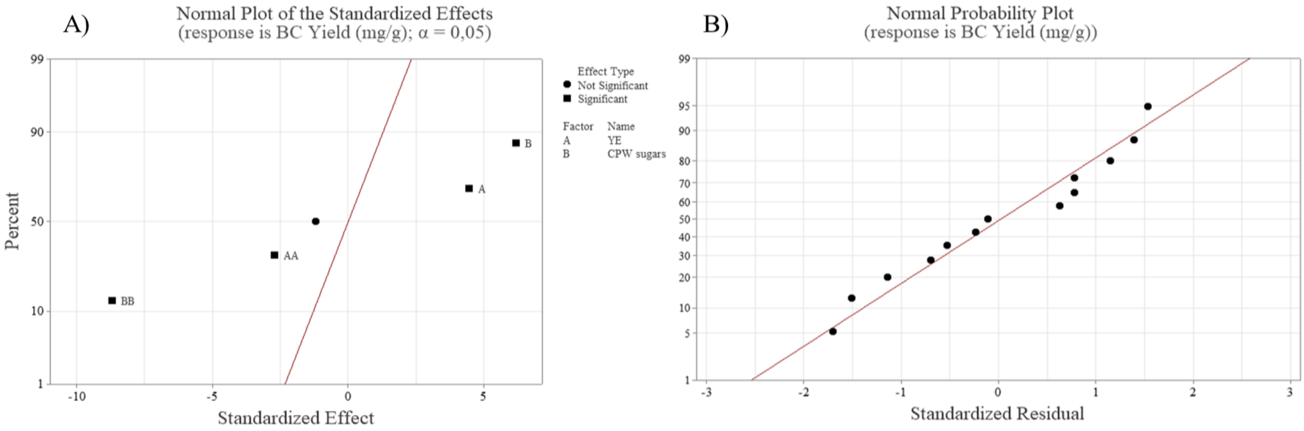

Figure S4. Response surface regression analysis of central composite design (CCD). A) Normal plot of the standardised effects showing the statistical significance of factor A (yeast extract (YE)), factor B (CPW sugars) and their quadratic effects on BC yield (g/g), chosen as model response; B) Normal probability plot showing that the internally studentized residuals closely follow a straight line, suggesting that the residuals are approximately normally distributed.

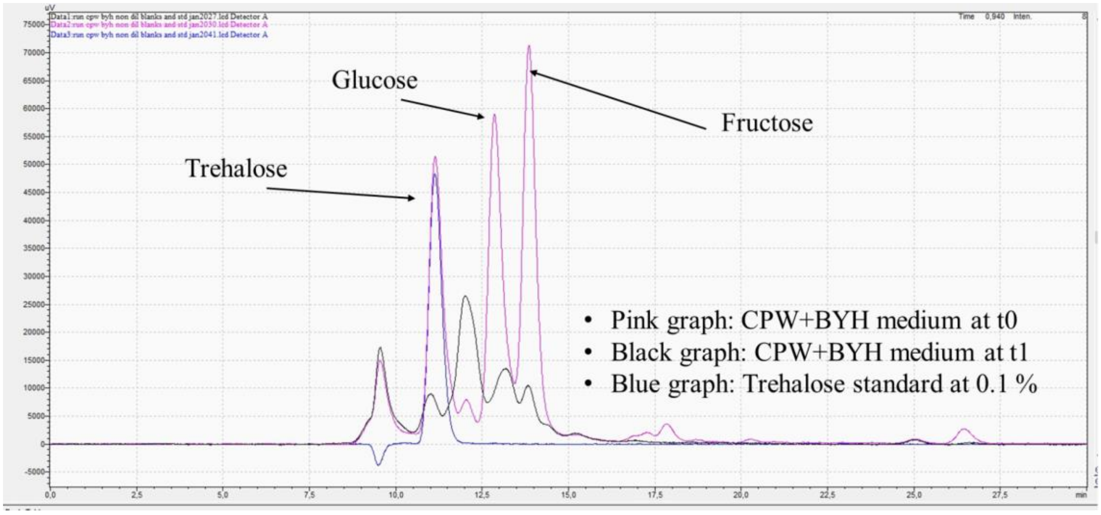

Figure S5. HPLC elution graphs of the CPW-BYH-based medium before (pink graph, t0) and after (black graph, t1) *K. sucrofermentans* cultivation. The blue graph representing the trehalose standard highlights its presence in the CPW-BHY-based medium. Due to the overlapping of trehalose and sucrose peaks, it is not possible to quantify their concentration precisely.
